# The Influence of Leisure Activity Types and Involvement Levels on Leisure Benefits in Older Adults

**DOI:** 10.3389/fpubh.2021.659263

**Published:** 2021-07-14

**Authors:** Jintian Li, Baogen Zeng, Peiyan Li

**Affiliations:** ^1^College of Physical Education, Shaoguan University, Shaoguan, China; ^2^School of Politics and Law, Shaoguan University, Shaoguan, China

**Keywords:** older adults, guangdong, leisure type, leisure participation, leisure benefits

## Abstract

**Purpose:** To explore the types of leisure activities, the degree of activity involvement, and the relationship between leisure benefits in older adults in cities and towns in Northern Guangdong, China; to provide valuable references for formulating related policies on the leisure industry for relevant governing bodies and for gaining the optimal benefits for industry business people and leisure enthusiasts.

**Methods:** After searching for a large amount of literature and expert consultations, a questionnaire on the leisure involvement and leisure benefits scale was constructed. The participants aged 60 years or older were recruited as the survey population. By using SPSS statistics 21.0, the raw and processed data in this study were analyzed and interpreted.

**Results:** (1) The approach that the leisure involvement levels were divided into subjective and objective involvement levels to predict leisure benefits was more comprehensive and reliable than uni-level prediction. Among them, the overall leisure involvement level had a significant positive effect on the leisure benefits of leisure participants; the performance of the subjective involvement level to enhance the leisure benefits comprehensively surpassed the objective involvement level. (2) The effect of leisure involvement levels on leisure benefits was affected by the type of leisure, which was manifested as physiological and psychological leisure pleasure; the degree of leisure involvement had an inverted U-shaped relationship with its emotional leisure benefits. For individuals with physiological hedonistic leisure, the degree of leisure involvement had an inverted U-shaped relationship with its emotional, social, and self-realized leisure interests. (3) For individuals with psychological leisure, the degree of leisure involvement was positively U-shaped with its social and self-affirmed leisure benefits. However, this finding needs to be confirmed by further research.

**Conclusion:** There are two types of linear and curvilinear relationships between the degree of leisure involvement and leisure benefits, which makes the connection between leisure involvement and leisure benefits deviate from the linearity in a particular situation and present an inverted U-shaped or positive U-shaped relationship, which shows, when the individuals are under- or over-volume leisure activities involvement, they will not create excellent leisure benefits.

## Introduction

Leisure activitiesrefer to activities in which individuals participate in their free time outside of their mandatory time (such as work, class, and sleep). It is an action based on an open consciousness, free choice, and self-determination, and obtained from the improvement of the sense of implication and experience of the activities, such as reading, sports, climbing, social activities, chatting, or shopping (1, 2). Leisure experience is the process in which individuals spend on leisure activities they are involved in. People regard leisure as essential to cultivate temperament and self-development the more frequently they participate in these activities. Leisure activities can improve the physical and mental health of individuals and have the significance of regulating the body and mind, alleviating the stress of life, and providing a pleasant experience (3, 4). Benefits of leisure activities include stress reduction, relaxation through participation in pleasant experiences, and the creation of new social relationships by participation in leisure activities (5).

Previous studies showed that participation in leisure activities could bring multiple benefits to individuals. These findings are on the topics of physical, psychological, social, and self-development benefits, such as leisure for personal and mental development (6, 7), health promotion (8, 9), family relationships (10, 11), interpersonal interaction (12, 13), and economic benefits (12, 14). Some studies have suggested that the level of participation in leisure activities is a measure of the physical and mental health of an individual (3). However, when looking at the findings of leisure participation of scholars and leisure interests at home and abroad, there are many inconsistent conclusions. Some scholars (14, 15) found that the more frequent the individuals participate in a specific leisure activity, the more leisure benefits they can be obtained, while another study found that there is no positive relationship between leisure participation and leisure benefits (10). So, does the level of leisure involvement predict the benefits of leisure? Do the leisure involvements and benefits have a positive linear relationship or a curved (i.e., U-shaped) relationship? In other words, is participation in leisure activities proportional to leisure benefits to a specific range of involvement? When leisure involvement exceeds a certain degree, does this positive linear relationship still hold?

Based on the above considerations, this study proposed two assumptions: (1) all leisure activities can bring diversified leisure benefits to individuals; (2) the degree of participation of individuals in leisure activities can only create more leisure benefits for themselves. In order to test these two assumptions, this study also innovated method in data processing. First, the individual leisure activities were classified, and then two regression analyses (linear and curve) methods were used to explore the involvement of the classified leisure activities. Whether a specific leisure activity can create specific leisure benefits, second, because previous studies represented leisure involvement by participating in leisure or performing a particular leisure activity, leisure frequency does not fully cover the leisure activities of individuals. The overall level of involvement in leisure activities of individuals may include watching TV an hour per day and participating in yoga training two times a week. Yoga training is more active than watching TV, but watching TV is funnier than yoga training. In order to amend the degree of leisure involvement, this study takes the objective participation of individuals in the frequency and subjective involvement degree in leisure activities separately as two variables to independently evaluate the degree of leisure involvement.

## Methods

### Participants

Participants aged ≥60 years old were selected as the investigation population. A total of 2,000 questionnaires were issued in fitness clubs, fitness venues, street fitness trails, and park morning training venues. After collecting and processing questionnaires, 1,893 questionnaires were valid in this study, including 451 samples from fitness clubs, 569 from fitness venues in the community, 425 from street fitness trails, and 448 from morning exercise venues in the park.

Each participant provided written informed consent and was assured confidentiality. This survey followed local law and regulation and has been approved by the Social Science Foundation of Guangdong Province (No. GD17CTY02).

On completion, the preliminary analysis showed that the age of the respondents ranged from 60 to 86 years old (M = 68.5, SD = 10.4); among them, 1,072 were women (57%) and 821 were men (43%).

### Instruments

#### Questionnaire Design

To comprehensively understand the leisure activities and leisure benefits on older adults in Northern Guangdong, this study developed the “Leisure Activities Involvement Status and Leisure Benefits Scale.” This scale consists of three components as follows:

1. The demographic information.
2. For this component, some necessary demographic information, such as gender, age, monthly income, occupation, education, requires the respondents to fill in.
3. The leisure involvement scale
4. Referring to the findings of previous scholars (4, 16, 17), this study used the 5-point Likert scale to comprehensively quantify the degree of leisure involvement from two dimensions: Dimension 1: Subjective involvement, consisting of six items; Dimension 2: Objective involvement, including three items, using Fox (18) calculation formula of activity intensity: objective involvement degree = exercise frequency × (duration + average intensity).
5. The leisure benefits scale
6. Leisure benefits are the reason for continuing to participate in leisure. It refers to the benefits of physical improvement or meeting the personal needs of individuals in the process of participating in leisure activities (9, 19). Individuals can also temporarily escape the heavy burden, work, and interpersonal constraints by leisure activities, and then get ideal personal compensation or new relationships from leisure participation. Based on the previous research design (20, 21), 22 items of leisure benefits were constructed in this study.

#### The Validity and Reliability Test of the Questionnaire

1. The leisure involvement scale of the resident contains 11 items. After factor analysis, two common factors were extracted by the principal axis method. The results showed that the KMO = 0.84 and Bartlett's spherical test values were significant (*P* < 0.001), indicating that it was very suitable for factor analysis, and the cumulative interpretation of the two common factors reached 58.11%. After the load value analysis by rotation, two original items were eliminated because the factor loading values were low. The first common factor contains six items, e.g., “The leisure activity is vital to me”; “The leisure activity is closely related to me”; “The leisure activity is of great significance to me”; “I will spend a lot of effort to collect information about this leisure activity.” The above items are mainly related to the importance of leisure activities to the participants, the relevance to daily life, attractiveness, etc. The subjective perception of the participants was involved in leisure activities, so it was named the “subjective involvement level” factor, which has a contribution rate of 34.11%; the second common factor contains three items, e.g., “How long do you spend on casual physical exercise?”; “How many times do you do leisure sports a week?”; and “The intensity of each time you participate in leisure sports exercise.” Those are mainly related to leisure sports activity frequency, intensity, and time allocation, reflecting the leisure practice of the participants. It was named the “objective involvement level” factor, and its contribution rate was 24.00%. The internal consistencies of the two common factors Cronbach's α coefficients were 0.89 and 0.85. It shows that the measurement has better reliability.
2. The leisure benefits scale contains four constructs made up of 17 items. They are listed below:

Health benefit:

1. Leisure activities can increase physical strength and exercise physical fitness;
2. leisure activities can help improve healthy physical fitness;
3. leisure activities can prevent or control diseases;
4. leisure activities can eliminate fatigue and restore physical strength.

Emotional benefits:

1. Participating in leisure activities can relax tense emotions;
2. participating in leisure activities can enhance psychological satisfaction;
3. leisure activities can ease the pace of life;
4. leisure activities can dispel life boredom.

Social benefits:

1. Leisure activities can strengthen the connection between my friends and me;
2. leisure activities help to meet new friends;
3. participating in leisure activities helps to establish interpersonal relationships;
4. participating in leisure activities can increase the connection with others.

Self-fulfillment interests:

1. Engaging in leisure activities helps to train work problem-solving skills of an individual;
2. participating in leisure activities helps to stimulate the potential of an individual;
3. the sense of accomplishment given by leisure activities can relatively boost the sense of work achievement;
4. participating in leisure activities helps to improve the self-confidence of an individual; and
5. participating in leisure activities can give a sense of accomplishment or self-challenge enjoyment.

The exploratory factor analysis was performed. Four common factors were extracted by the principal axis method. The KMO = 0.87 and Bartlett's spherical test value were significant (*P* < 0.001), indicating that they were suitable for factor analysis, and the cumulative interpretation of the four common factors reached 83.33%. After analysis of the load value after rotation, five items in the original scale were excluded because the load value was too low. Among them, the first common factor contains four items, which are mainly related to the benefits of physical strength, leisure fitness, prevention and disease control, and elimination of job burnout from leisure activities of participants, so they have named the “health benefit” factor, which has the highest contribution rate (28.86%); the second common factor contains four items, which are mainly related to the benefits of the participants, such as emotional relaxation, psychological gratification, relaxation of the pace of life, and the elimination of boredom from leisure activities. Therefore, it has named as the “emotional benefit” factor, and its contribution rate was 25.53%; the third common factor contains four items, and its content mainly relates to the ability of the subjects to strengthen the connection with friends, meet new friends, establish interpersonal relationships, and increase the opportunities for contact with others, so it is named the “social benefit” factor, which contributes 16.16%; the fourth common factor contains five items, which mainly involve the participants from leisure activities, getting corresponding help, such as solving job problems, inspiring self-potential, boosting personal work achievement, gaining self-confidence, and having self-challenge fun, so it was named the “self-realization benefit” factor, and its contribution rate is 12.78%. The Cronbach's α coefficients of the four common factors were 0.81, 0.84, 0.79, and 0.83, respectively. It showed that the measurement has good reliability. For more details, see Table 1.

**Table 1:** Extraction of common factors and reliability analysis of leisure involvement and leisure benefit scale in Northern Guangdong.

**Scales**	**KMO and Bartlett test**	**Common factor naming**	**Items**	**Eigenvalue**	**Explained variance%**	**Accumulated explained variance%**	**Cronbach α**
DLI	KMO = 0.84: *P* <0.001	DOI	6	9.58	34.11	34.11	0.89
		DSI	3	6.74	24.00	58.11	0.85
LB	KMO = 0.87: *P* <0.001	HB	4	7.25	28.86	28.86	0.81
		EB	4	5.66	25.53	54.39	0.84
		SB	4	4.06	16.16	70.55	0.79
		SRB	5	3.21	12.78	83.33	0.83

*DLI, Degree of leisure involvement; LB, leisure benefits; DSI, degree of subjective involvement; DOI, degree of objective involvement; HB, health benefits; EB, emotional benefits; SB, social benefits; SRB, self-realization benefits*.

### The Definitions of Types of Leisure Activities

According to definitions of some scholars of different types of leisure activities (22–25), this study proposed a two-dimension model: the first dimension is called “physiological leisure,” which means that the activities selected by leisure participants are mainly to consume physical resources (i.e., consuming physical exertion). Such leisure activists need to perform physical activities, such as sports, outdoor, and adventure activities; the second dimension is called “psychological leisure,” which means that the activity participants are executed by consuming the spiritual resources of the individuals, such as knowledge-based, literary, and social activities. On the other hand, from the perspective of motivating leisure benefits, whether individuals choose physiological leisure or psychological leisure activities will produce two types of results: pragmatic and hedonic benefits. Based on the theoretical and practical consequences of these scholars, this study classifies leisure types into four categories:

Category A: physiological and pragmatic leisure. Individuals participate in such exercises to pursue practical benefits, such as outdoor activities (e.g., mountain climbing, hiking), adventure activities (e.g., rafting, rock climbing, survival games), and tour activities (travel or journey).
Category B: physiological and hedonic leisure. Such leisure activities are also mainly performed by physical abilities. Individuals participate in such exercises to pursue hedonic benefits, such as sports activities (e.g., playing a ball, running, yoga) and rest activities (e.g., shopping, various dances).
Category C: psychological and hedonic leisure; Such leisure activities are mainly performed by mental or brain abilities. Individuals perform this activity to pursue pragmatic benefits, such as knowledge (e.g., learning, talent class: calligraphy, Go, ceramic art DIY) and literary activities (e.g., reading, painting, music, exhibition, and a lecture).
Category D: psychological and hedonic leisure. Such leisure activities are also mainly performed by mental or brain abilities. Individuals that perform such activities will obtain hedonic benefits, such as social activities (e.g., dinner, networking, chat, and playing cards) and mass media activities (e.g., TV, movies, radio, virtual games, and networks).

### Statistical Analyses

SPSS Statistics for Windows 21.0 was used to process all data indicators. Linear and curve regression models were mainly used to explore the relationship between leisure activity involvement and leisure benefits. The significant level of all indicators was set to α = 0.05.

## Results

### The Impact of Leisure Involvement on the Benefits of Physiological and Pragmatic Leisure

Table 2 shows the following:

1. Only the linear model can explain the impact of objective involvement on physiological and practical leisure benefits, and its four curve models are not statistically significant. From the characteristics of the linear structure model, it can be seen that the linear regression coefficient of the degree of objective involvement in the health and leisure benefits is positive (Beta = 0.271, *P* = 0.029 <0.05, *R*^2^ = 0.129), indicating that it has a significant positive effect, and the degree of objective involvement can explain the 12.9% variation in health leisure benefits; the degree of objective involvement affects emotional leisure benefits (Beta = −0.034, *P* = 0.412 >0.05), social leisure benefits (Beta = 0.079, *P* = 0.304 >0.05), and self-realization of leisure benefits (Beta = 0.067, *P* = 0.339 >0.05) both had no linear relationship.
2. Of the effects of subjective involvement on physiological and pragmatic leisure benefits, all four linear models are statistically significant. Still, only one of the four curve models is statistically significant. The characteristics of the linear model: the degree of subjective involvement in health leisure benefits (Beta = 0.512, *P* < 0.001, *R*^2^ = 0.247), emotional leisure benefits (Beta = 0.587, *P* < 0.001, *R*^2^ = 0.391), social leisure benefits (Beta = 0.641, *P* < 0.001, *R*^2^ = 0.409), and self-realization leisure benefits (Beta = 0.606, *P* < 0.001, *R*^2^ = 0.368) have significant positive effects. Its linear coefficient r is positive, of which the social leisure benefit model has the highest explanatory power (40.9%), and the health leisure benefit model has the smallest explanatory power (24.7%). From the four curve models, only one model has statistical significance. The effect of subjective involvement on emotional leisure benefits has a non-linear effect (the coefficient of square term Beta = −1.325, *P* = 0.017 <0.05, *R*^2^ = 0.436). Obviously, after using curve regression, the degree of subjective involvement, explaining the emotional leisure benefits, is 43.6%, which exceeds the linear regression explanation amount of 39.1%. However, the value of the squared coefficient r is negative, indicating that the parabola opening is downward. Therefore, it can be inferred that the subjective involvement in emotional benefits has an inverted U-shaped relationship.

**Table 2:** The effects of the degree of leisure involvement on physiological pragmatic leisure benefits in older adults.

**Variables**	**Health benefit**		**Emotional benefit**		**Social benefit**		**Self-realization benefit**	
	**Linear**	**Curve**	**Linear**	**Curve**	**Linear**	**Curve**	**Linear**	**Curve**
SI	0.271^*^	0.307	−0.034	0.187	−0.079	−0.216	−0.067	0.361
SI^2^		−0.138		−0.224		0.154		−0.305
*R*^2^	0.129	0.135	0.007	0.005	0.008	0.017	0.006	0.018
Test	0.029	0.726	0.412	0.506	0.304	0.704	0.339	0.405
OI	0.512^**^	1.427^**^	0.587^**^	1.924^***^	0.641^**^	1.405^**^	0.606^**^	1.047^**^
OI^2^		−0.902		−1.325^***^		−0.761		−0.424
*R*^2^	0.247	0.281	0.391	0.436	0.409	0.427	0.368	0.323
Test	0.000	0.106	0.000	0.017	0.000	0.128	0.000	0.417

*OI, objective involvement; SI, subjective involvement; R^2^, coefficient of determinant;*

* *p <0.05*;

** *p <0.01*;

*** *p <0.001*.

### The Impact of Leisure Involvement on the Benefits of Physiological and Hedonistic Leisure

Table 3 shows:

1. The impact of objective involvement on the benefits of physiological and hedonic leisure could only be explained in a linear modal, as the four curve models were not statistically significant. From the perspective of the influence of the linear model, the degree of objective involvement in health leisure benefits (Beta = 0.329, *P* = 0.003 <0.01, *R*^2^ = 0.114), emotional leisure benefits (Beta = 0.231, *P* = 0.033 <0.05, *R*^2^ = 0.070), and self-realization leisure benefits (Beta = 0.287, *P* = 0.006 <0.01, *R*^2^ = 0.088) had a significant positive effect (each Beta was positive), while social leisure benefits (Beta = −0.230, *P* = 0.024 <0.05, *R*^2^ = 0.066) had a significant negative effect (Beta was negative); from the perspective of explanatory power, the health leisure benefit model was the highest (11.4%), and the social leisure benefit model was the lowest (6.6%).
2. The impact of subjective involvement on the benefits of physiological and hedonic leisure can be explained not only by the linear model, but, except the health leisure benefits, the remaining three-dimensional benefits can be better explained from the curve model. In terms of the linear model, the subjective level of involvement in health leisure benefits (Beta = 0.597, *P* <0.001; *R*^2^ = 0.369), emotional leisure benefits (Beta = 0.481, *P* < 0.001; *R*^2^ = 0.233), social leisure benefits (Beta = 0.629, *P* < 0.001; *R*^2^ = 0.378), and self-realization leisure benefits (Beta = 0.677, *P* < 0.001; *R*^2^ = 0.459) were significantly positive effect, of which the self-realization benefit model had the highest explanatory power (45.9%), and the emotional leisure benefit model had the lowest explanatory power (23.3%). In terms of the curve model, subjective involvement and emotional leisure benefits (Beta = −1.335, *P* = 0.029 <0.05; *R*^2^ = 0.264), social leisure benefits (Beta = −1.239, *P* = 0.008 <0.01; *R*^2^ = 0.391), and self-realizing leisure benefits (Beta = −1.085, *P* = 0.019 <0.05; *R*^2^ = 0.511) were significantly better than the explanatory power of the linear model (the curve regression *R*^2^-values were greater than their corresponding linear regression *R*^2^-value). In addition, the signs of the coefficients of the square items of the independent variables of the three curve models were negative, indicating that the parabola opens downward, showing that the subjective involvement had an inverted U-shaped relationship with emotions, society, and self-realization benefits.

**Table 3:** The impact of degrees of leisure involvement on physiological and hedonistic leisure.

**Variables**	**Health benefit**		**Emotional benefit**		**Social benefit**		**Self–realization benefit**	
	**Linear**	**Curve**	**Linear**	**Curve**	***c***	**Curve**	**Linear**	**Curve**
SI	0.329^*^	0.084	0.231^**^	1.049	−0.230^**^	0.291	0.287^**^	−0.406
SI^2^		0.236		−0.827		−0.136		0.715
*R*^2^	0.114	0.105	0.070	0.056	0.066	0.039	0.088	0.066
Test	0.003	0.623	0.033^*^	0.309	0.024^*^	0.908	0.006	0.410
OI	0.597^**^	1.356^**^	0.481^**^	1.655^**^	0.629^***^	1.881^***^	0.677^***^	1.677^***^
OI^2^		−0.761		−1.335^**^		−1.239^***^		−1.085^**^
*R*^2^	0.369	0.393	0.233	0.256	0.378	0.391	0.459	0.504
Test	0.000	0.110	0.000	0.029	0.000	0.008	0.000	0.019

*OI, objective involvement; SI, subjective involvement; R^2^, coefficient of determinant*;

* *p <0.05*;

** *p <0.01*;

*** *p <0.001*.

### The Impact of the Degree of Leisure Involvement on the Benefits of Psychological and Pragmatic Leisure

Table 4 shows:

1. Only the linear model can explain the impact of the degree of objective involvement on the benefits of psychological and pragmatic leisure because the four curve models were not statistically significant; only two of the four linear models have reached a significant level. The objective involvement degree had significant effects on emotional leisure benefits (Beta = 0.282, *P* = 0.005 <0.01; *R*^2^ = 0.087) and self-realization leisure benefits (Beta = 0.244, *P* = 0.021 <0.05; *R*^2^ = 0.083). It had no positive effect on health leisure (Beta = 0.015, *P* = 0.691 >0.05) and social leisure benefits (Beta = 0.029, *P* = 0.785 >0.05).
2. The impact of personal involvement on the benefits of psychological and pragmatic leisure could be explained by linear models, and curve models could better explain the social and self-realization benefit. From the linear model, the subjective level of involvement in health leisure benefits (Beta = 0.244, *P* = 0.016 <0.05; *R*^2^ = 0.071), emotional leisure benefits (Beta = 0.434, *P* < 0.001; *R*^2^ = 0.207), social leisure benefits (Beta = 0.311, *P* = 0.024 <0.05; *R*^2^ = 0.097), and self-realization leisure benefits (Beta = 0.510, *P* < 0.001; *R*^2^ = 0.268); all the impacts were positive. It showed that the self-realization leisure benefit model had the highest explanatory power (26.8%), while the health leisure benefit model had the lowest explanatory power (7.1%). From the curve regression model, subjective involvement and social leisure benefits (Beta = 1.901, *P* = 0.028 <0.05; *R*^2^ = 0.169) and self-realization leisure benefits (Beta = 1.663, P = 0.021 <0.05; *R*^2^ = 0.312), and the explanatory power (16.9 and 31.2%) was significantly higher than their corresponding linear models (9.7 and 26.8%). In addition, both the signs of the square item coefficients r of the two curve models were positive, indicating that the opening of the parabola was upward. It could be seen that the degree of personal involvement had a positive U-shaped relationship with social leisure benefits and self-realization benefits.

**Table 4:** The impact of degrees of leisure involvement on psychological and pragmatic leisure.

**Variables**	**Health benefit**		**Emotional benefit**		**Social benefit**		**Self–realization**	
	**Linear**	**Curve**	**Linear**	**Curve**	**Linear**	**Curve**	**Linear**	**Curve**
OI	0.015	0.809	0.282^***^	0.349	0.029	−0.625	0.244^**^	1.325
OI^2^		−0.745		−0.034		0.723		−1.234
*R*^2^	0.000	0.018	0.087	0.091	0.000	0.018	0.083	0.068
Test	0.691	0.401	0.005	0.854	0.785	0.309	0.021	0.135
SI	0.244^**^	−0.425^*^	0.434^***^	−0.742	0.311^***^	−1.614^*^	0.510^***^	−1.201
SI^2^		0.741		1.223		1.901^**^		1.663^**^
*R*^2^	0.071	0.074	0.207	0.219	0.097	0.169	0.268	0.312
Test	0.016	0.411	0.000	2.214	0.024	0.028	0.000	0.021

*OI, objective involvement; SI, subjective involvement; R^2^, coefficient of determinant*;

* *p <0.05*;

** *p <0.01*;

*** *p <0.001*.

### The Impact of the Involvement Degree on the Benefits of Psychological and Hedonic Leisure

Table 5 shows the following:

1. Of the impact of objective involvement on the benefits of psychological and hedonic leisure, only one of the four linear and four curve models reached a significant level; it showed that the degree of objective involvement had a negative linear effect on the health benefits (Beta = −0.170, *P* = 0.037 <0.05; *R*^2^ = 0.072); and the degree of objective involvement had no linear impact on emotional benefit, but there was a curve impact (Beta = −2.322, *P* = 0.021 <0.05; *R*^2^ = 0.088), because the coefficient of the square item was negative, indicating that there was an inverted U-shaped non-linear relationship between the involvement degree and emotional benefit.
2. The effect of personal involvement on the benefits of psychological and hedonic leisure could only be explained by linear models, of which the four linear models had statistical significance. From the perspective of the model structure, the degree of subjective involvement in health leisure benefits (Beta = 0.283, *P* = 0.003 <0.01; *R*^2^ = 0.011), emotional leisure benefits (Beta = 0.527, *P* = 0.000 <0.001; *R*^2^ = 0.289), social leisure benefits (Beta = 0.265, *P* = 0.006 <0.01; *R*^2^ = 0.085), and self-realization leisure benefits (Beta = 0.608, *P* < 0.001; *R*^2^ = 0.365); each r was positive. Among them, the self-realization leisure benefit model had the highest explanatory power (36.5%), and the social leisure benefit model had the lowest explanatory power (8.5%).

**Table 5:** The impact of degrees of involvement on psychological and hedonic leisure.

**Variables**	**Health benefit**		**Emotional benefit**		**Social benefit**		**Self–realization benefit**	
	**Linear**	**Curve**	**Linear**	**Curve**	**Linear**	**Curve**	**Linear**	**Curve**
OI	−0.170*	−1.308	−0.138	2.274	−0.158	−2.314	0.038	0.576
OI^2^		1.266		−2.322*		1.775		−0.514
*R*^2^	0.072	0.044	0.027	0.088	0.028	0.049	0.001	0.003
Test	0.037	0.431	0.186	0.021	0.126	0.168	0.761	0.602
SI	0.283***	0.523	0.527***	0.035	0.265***	0.712	0.608***	0.275
SI^2^		−0.274		0.481		−0.429		0.339
*R*^2^	0.011	0.085	0.289	0.276	0.085	0.078	0.365	0.368
Test	0.003	0.713	0.000	0.478	0.006	0.442	0.000	0.626

*OI, objective involvement; SI, subjective involvement; R^2^, coefficient of determinant*;

*p <0.05;

**p <0.01;

***p <0.001.

## Discussion

The goal of this study was to reveal the relationship between leisure involvement and leisure benefits. As leisure activities vary, their differences are bound to interfere with the relationship between leisure involvement and leisure benefits. This study first confirmed the type of leisure participation of individuals and whether the leisure involvement of different forms of leisure helps enhance leisure benefits. Based on previous literature, this study proposed two binary structural conditions based on exercises physiology, psychology, and leisure values (17, 23–26): (1) to determine whether an individual is using physical (physiological) or psychological resources to perform leisure activities; (2) to determine the leisure pursuit of leisure value (the fundamental motivation of the activity), that is, whether the leisure benefits obtained by the people participating in leisure activities are actual needs or emotional expressions. Based on the above discussion, leisure activities were classified into four categories: physiological and pragmatic (such as outdoor, adventure, and tourism), physiological and hedonic (such as sports and rest), psychological and pragmatic (such as knowledge and arts), and psychological and hedonic (such as social and mass media). This practical and straightforward binary classification architecture not only included multiple types of leisure activities but also verified the rationality of this classification from empirical results, thereby making up for the lack of a subjective classification of many scholars in the past and suggesting that this classification be promoted in the future research.

Based on a large amount of literature, the survey scale for the degree of leisure involvement and leisure benefits of urban residents in northern Guangdong constructed by this study has good measurement validity. However, previous scholars adopted either an objective evaluation method for leisure involvement, which is based on the frequency of individual participation in leisure activities, or a subjective observation evaluation method based on the internal needs and values of individuals, importance, and benefits to measure involvement. These two methods have their one-sidedness in assessing the degree of leisure involvement. Notably, it is unfair to objectively evaluate the degree of leisure involvement only by the frequency of participation. However, this study combined the two methods. It explicitly affirmed leisure activity as an objective evaluation method of leisure involvement, thereby making the evaluation of leisure involvement more comprehensive and reliable. A large number of previous studies should confirm that no matter what type of leisure, with the increase of leisure involvement, leisure participants have a significant positive effect on leisure benefits (22, 27–30). However, this study found that leisure activity should restrict the role of leisure involvement in enhancing leisure benefits, and the positive effect of leisure involvement on leisure benefits is only correct to a certain extent. Excessive or insufficient involvement may be harmful to the promotion of leisure interests.

This study found that, for the participants in physiological and pragmatic leisure activities, the impact of subjective involvement on their leisure benefits was the most important. It had a comprehensive effect on improving health, emotions, society, and self-realization of leisure benefits of participants, while objective involvement degree can only contribute to health benefits; at the same time, this study finds that the impact of subjective involvement on emotional leisure benefits was not a linear effect but a significant inverted U-shaped relationship. This relationship means that, when subjective involvement of participants was excessive, their emotional leisure benefits would gradually decline. Previous research reported that proper participation in tourism could be fun and positive for self-life; proper participation in games could help relieve work pressure, but if an individual game was too severe or too concerned about the activities they were engaged in, it was no longer a game, because when physical resources of individuals were used too much, it may lead to physical fatigue, which made it impossible to continue working, thus reducing the leisure benefits (16, 31). These reports provided evidence for the findings of this study. In short, when physiological and pragmatic leisure is active, the leisure benefits of emotion may not belong to the value they seek. Therefore, when participating in physiological and pragmatic leisure activities, such as travel, mountain climbing, and rock climbing, the degree of involvement should be appropriate to achieve emotions. On the relief or relaxation, this study found no non-linear relationship between objective involvement and leisure benefits. In contrast, subjective involvement had significant inverted U-shaped relationships with emotional, social, and self-realization leisure benefits, which meant that the participants had excessive subjective involvement. As time went by, its leisure benefits will gradually decline. Piko and Vazsonyi (32) pointed out that shopping could not provide the participants with more profound experience and learning, which would cause the participants to feel bored or powerless. The reason was that excessive sports and rest-type leisure activities would cause emotional exhaustion. Shopping is a typical physiological and hedonic leisure activity, so some scholars have supported this finding in this study. Based on these understandings, individuals should be appropriately involved in physiological and hedonic leisure activities, such as playing basketball, yoga, and shopping, to relax tension, establish good interpersonal relationships, and improve self-confidence.

For the participants in psychological and pragmatic leisure activities, this study found that the degree of personal involvement had a more significant impact on leisure benefits than the degree of objective involvement. The degree of objective involvement only contributed to the improvement of emotional and self-realization leisure benefits. Still, the subjective involvement degree significantly improves the health, emotional, social, and self-realization leisure benefits. This study also found that the impact of subjective involvement on the social and self-realization benefits of psychological and pragmatic leisure was not linear but a significant positive U-shaped relationship. That is to say, when the degree of objective involvement increases, the social leisure and self-realization benefits of the leisure participants gradually decline and reach the lowest point, and then the degree of involvement increases and then increases rapidly. To date, these findings have not been supported by corresponding reports, but they had, precisely, the opposite relationship with the results of Liu et al. (33). The latter believed that when individuals were too much or too little to participate in arts and rest leisure activities (a typical psychological and pragmatic type), this activity would not help reduce the degree of emotional exhaustion but would make the emotional exhaustion more serious; Liu et al. (33) did not directly reveal this U-shaped relationship, but the results presented were said to be a typical inverted U-shaped relationship. The results obtained in this study were positive U-shaped relationships. The two works seem to be conflicts, but the authors thought it might be a different perspective to deal with the problem. From the view of this study, any leisure enthusiast will encounter a bottleneck from beginning to reaching more profound leisure, which means that he or she must experience setbacks during the leisure learning process. At this time, the deeper the involvement of leisure, the bigger this frustration should be, which leads to the decline of leisure benefits. As leisure enthusiasts continue to overcome difficulties and get out of the predicament, it may lead to the rapid improvement of leisure benefits, which is a positive U-shaped relationship. Finally, for the participants in leisure activities of psychological enjoyment, the impact of subjective involvement on leisure benefits becomes more prominent than the objective involvement level. The latter only affects health leisure benefits, and this influence is negative. The explanatory power of the model was also meager, and the degree of subjective involvement has significant positive effects on health, emotions, society, and self-realization of leisure benefits. However, among the participants in psychological and hedonic leisure, a study has found that the degree of objective involvement has a significant inverted U-shaped relationship to the emotional leisure benefits. Chao (34) found that, with the increase of leisure involvement, media participants have not shown significant positive benefits in personal self-growth, interpersonal interaction, or overall leisure perception benefits. Because media leisure activities are psychological and hedonic types, although the results of this scholar have not fully supported the findings of this study, at least it is inevitable that those who are excessively involved in this type of leisure activities will not enhance their leisure benefits. When individuals are in the proper frequency of participation, they can get more emotional leisure benefits. Therefore, when participating in psychological and hedonic leisure activities, such as dining, surfing on the Internet, and watching movies, the number of participants must not be too much or too little; otherwise, it will cause an emotional burden or dissatisfaction (35, 36).

### Limitations

In this study, during the data analysis, the population has been regarded as a whole. Thus, this study did not examine its age, income, or other demographic characteristics difference; it should be a further study in the future.

## Conclusions

The overall level of leisure involvement has a significant positive effect on its leisure benefits. The positive promotion of leisure benefits by subjective involvement levels is significantly higher than the objective involvement level, which means strengthening individual internal needs and value recognition. The perceptual relevance and the importance of the object are often more effective in improving leisure benefits than merely emphasizing the amount and frequency of leisure activity participation. For the participants in physiological and pragmatic leisure, the degree of leisure involvement has an inverted U-shaped relationship with their emotional leisure benefits. For the participants in physiological and hedonic leisure, the degree of leisure involvement has an inverted U-shaped relationship with their emotional, social, and self-realization leisure interests, which means that the degree of leisure activity involvement cannot be excessive or untimely. Otherwise, it will not create excellent leisure benefits.

## Data Availability Statement

The original contributions presented in the study are included in the article/supplementary material, further inquiries can be directed to the corresponding author/s.

## Ethics Statement

Ethical review and approval was not required for the study on human participants in accordance with the local legislation and institutional requirements. The patients/participants provided their written informed consent to participate in this study.

## Author Contributions

JL and BZ: conceptualization. JL: methodology, investigation, writing—original draft preparation, project administration, and funding acquisition. JL and PL: validation. BZ: formal analysis and writing—review and editing. All authors have read and agreed to the published version of the manuscript.

## Conflict of Interest

The authors declare that the research was conducted in the absence of any commercial or financial relationships that could be construed as a potential conflict of interest.
